# Texas women’s decisions and experiences regarding self-managed abortion

**DOI:** 10.1186/s12905-019-0877-0

**Published:** 2020-01-06

**Authors:** Liza Fuentes, Sarah Baum, Brianna Keefe-Oates, Kari White, Kristine Hopkins, Joseph Potter, Daniel Grossman

**Affiliations:** 1 Ibis Reproductive Health, 1736 Franklin St, Suite 600, Oakland, CA 94612 USA; 2 Steve Hicks School of Social Work, University of Texas at Austin, 1925 San Jacinto Blvd, Stop D3500, Austin, TX 78712 USA; 3 Population Research Center and the Department of Sociology, University of Texas at Austin, 305 E. 23rd Street, Stop G1800, Austin, TX 78712 USA; 4 Advancing New Standards in Reproductive Health (ANSIRH), Bixby Center for Global Reproductive Health, Department of Obstetrics, Gynecology and Reproductive Sciences, University of California, San Francisco, 1330 Broadway Suite 1100, Oakland, CA 94612 USA

**Keywords:** Abortion, Self-managed abortion, Self-induced abortion, Texas, Reproductive health, Reproductive rights, Pregnancy

## Abstract

**Background:**

Prior research has shown that a small proportion of U.S. women attempt to self-manage their abortion. The objective of this study is to describe Texas women’s motivations for and experiences with attempts to self-manage an abortion. The objective of this study is to describe Texas women’s motivations for and experiences with attempts to self-manage an abortion.

**Methods:**

We report results from two data sources: two waves of surveys with women seeking abortion services at Texas facilities in 2012 and 2014 and qualitative interviews with women who reported attempting to self-manage their abortion while living in Texas at some time between 2009 and 2014. We report the prevalence of attempted self-managed abortion for the current pregnancy among survey respondents, and describe interview participants’ decision-making and experiences with abortion self-management.

**Results:**

6.9% (95% CI 5.2–9.0%) of abortion clients (*n* = 721) reported they had tried to end their current pregnancy on their own before coming to the clinic for an abortion. Interview participants (*n* = 18) described multiple reasons for their decision to attempt to self-manage abortion. No single reason was enough for any participant to consider self-managing their abortion; however, poverty intersected with and layered upon other obstacles to leave them feeling they had no other option. Ten interview participants reported having a complete abortion after taking medications, most of which was identified as misoprostol. None of the six women who used home remedies alone reported having a successful abortion; many described using these methods for several days or weeks which ultimately did not work, resulting in delays for some, greater distress, and higher costs.

**Conclusion:**

These findings point to a need to ensure that women who may consider self-managed abortion have accurate information about effective methods, what to expect in the process, and where to go for questions and follow-up care. There is increasing evidence that given accurate information and access to clinical consultation, self-managed abortion is as safe as clinic-based abortion care and that many women find it acceptable, while others may prefer to use clinic-based abortion care.

## Background

Prior research has shown that a small proportion of US women attempt to self-manage their abortion. In a 2014 national survey of abortion patients, 2.2% had ever tried to end a pregnancy or bring back their period on their own [1]. In a 2014 representative survey of Texas women ages 15–49, 1.7% reported they had ever tried to end a pregnancy on their own [2]. Some studies have explored the context in which women choose to self-manage abortion. A 2008 qualitative study examining the abortion self-induction experiences of 30 women recruited from health care facilities in four US cities found that participants reported several reasons for choosing to attempt to self-manage an abortion, including being unable to afford the cost of clinic-based abortion care, wanting to avoid clinic-based care, and being young and therefore not knowing how or whether they could obtain a clinic-based abortion; others preferred self-induction because they thought it was easier or more natural [3]. The Texas survey found that women living in a county bordering Mexico, and who reported that they had ever found it difficult to obtain reproductive health services, for example because of high costs or lack of transportation, were more likely to report knowing someone who had attempted to self-manage an abortion or having done so themselves [2]. Women attempting to self-manage their abortion report using a range of methods, including herbs and vitamins [1, 3, 4], birth control pills [3], various food products [3], alcohol or drugs [4], and misoprostol/Cytotec [1, 3, 4].

Since 2011, Texas has implemented a series of laws restricting access to clinic-based abortion services. In 2011, Texas passed House Bill 15 (HB 15), which required women living less than 161 km (100 miles) from the nearest abortion facility to make an in-person visit at least 24 h before the abortion procedure for an ultrasound. These restrictions led to added burdens on women obtaining abortion care, including additional travel and negative emotional effects from the imposed waiting period [5].

In 2013, Texas passed House Bill 2 (HB 2), another restrictive abortion law. The law required physicians providing abortions to have admitting privileges at a hospital within 48 km (30 miles) of the facility, required them to administer medication abortion according to the U.S. Food and Drug Administration (FDA)-approved label for mifepristone, which was an outdated regimen at the time, banned most abortions after 20 weeks post-fertilization (22 weeks from the last menstrual period), and required facilities providing abortion to meet the standards of ambulatory surgical centers (ASCs). By November 1, 2013, the first three provisions of the law were enforced, resulting in one-third of abortion facilities closing immediately due the inability of providers to obtain hospital admitting privileges. In the first 6 months after HB 2 was enforced, the number of abortions performed in Texas declined 13% compared to the same period 1 year prior. The number of medication abortions provided, specifically, declined 70%, likely because the mandated protocol restricted the gestational length limit for medication abortion and required women to make an additional in-person visit to the facility [6].

Because of these barriers, some women who were unable to obtain an abortion in Texas after these restrictions were enforced may have traveled out of state for abortion care or had to carry their pregnancies to term. It is possible that increased travel required after HB 15’s two-visit requirement was enforced, as well as the drastic reduction in licensed abortion facilities and availability of medication abortion after HB 2, influenced some women to consider or attempt to self-manage their abortion. In a qualitative interview study with women seeking abortion services in Texas after HB 2, five of 23 respondents said they had thought about or looked into trying to self-manage their abortion; they said they did not pursue that option because they were worried that it would not be safe or that it would not be effective [7].

The objective of this study is to estimate the prevalence of attempted self-managed abortion among Texas abortion patients and describe Texas women’s motivations for and experiences with attempts to self-manage an abortion during the time periods in which HB 15 and HB 2 were debated, passed, and implemented. We report results from two data sources: two waves of surveys with abortion patients at Texas facilities and qualitative interviews with women who reported attempting to self-manage abortion while living in Texas at some time between 2009 and 2014.

## Methods

### Survey data collection and analysis

This analysis includes data from cross-sectional surveys conducted in 2012 and 2014 with patients seeking abortion care at facilities in Texas. The 2012 abortion patient survey was conducted between August and December in eight abortion facilities located in Austin, Dallas, El Paso, Houston, McAllen and San Antonio. The 2014 abortion patient survey was administered between May and August in ten facilities in Austin, Dallas, Fort Worth, Houston, and San Antonio. The clinic in McAllen where we collected data in 2012 was closed at the time of data collection in 2014. Detailed methods of the 2014 survey have been previously reported [8].

All clinics performed at least 1500 abortions per year, provided procedures up to a gestation of at least 14 weeks since last menstrual period (LMP), and were purposively sampled to include Planned Parenthood and independent clinics located across the state. For the 2012 survey, clinics had to offer both medication and surgical abortion, but that was not an inclusion criterion for the 2014 survey since many clinics stopped providing medication abortion [6]. Study staff recruited participants at each site for three to 6 days, depending on clinic schedule and volume. Every woman in the clinic waiting room was invited to participate in the survey. In the 2014 survey, at one facility clinic staff invited women to participate following their initial consult and interested women were directed to the project coordinator. Women were eligible to participate if they were seeking an abortion at the facility, were ≥ 18 years old, spoke English or Spanish, and had completed their pre-abortion ultrasound consultation visit. Eligible participants could complete the survey at ultrasound, procedure, or follow-up visits [8]. An information sheet describing the study purpose, procedure, risks, and benefits was offered to each survey respondent and a signed copy was obtained from them before completing the survey as part of the informed consent procedure. Survey participants received a $20 gift card as remuneration.

The survey included questions about socio-demographic characteristics, reproductive history, and access to abortion care. Most survey questions included in this analysis were identical in both years; we have indicated any questions that were worded differently. The main outcome variable was reporting an attempt to self-manage an abortion for the current pregnancy. Women were asked: “Did you take or do anything on your own to try to end this pregnancy or bring back your period this time before you came to this clinic for the abortion?” Women who responded “yes” to this question were then asked what they did to do so, and could answer: Misoprostol or Cytotec, some other drug or medication, herb, I hit myself or asked someone else to hit me in the abdomen,^1^ or something else (with a space for an open-ended response).

We merged data from the 2012 and 2014 surveys, and used Fisher’s exact tests to estimate significant differences between survey year samples at a critical value of *P* < 0.05. We then calculated the proportion of respondents for each year and the full sample that reported attempting to self-manage abortion. We also used Fisher’s exact tests to estimate significant differences between respondents who reported abortion self-induction and those who did not.

### Qualitative interview data collection and analysis

From October 2014–October 2015, we conducted qualitative, semi-structured interviews with women in Texas about their experiences with abortion self-induction. Women who could speak English or Spanish, were aged 18 years or older, and reported a history of abortion self-induction while living in Texas within the previous 5 years were eligible for the interview. Participants were recruited from those who reported a history of abortion self-induction in two studies: 1) the 2014 abortion patient survey described above and 2) a mixed-methods study comprising a survey and ethnographic study of the community, health service, and individual contexts for abortion access in the Lower Rio Grande Valley (LRGV). The LRGV, the southernmost region of Texas bordering Mexico, comprises four of the state’s ten lowest-income counties. Following HB 2, all abortion providers in the LRGV area had closed and the nearest clinic was in San Antonio, about 400 km (250 miles) away. A trained ethnographer conducted key informant interviews with LRGV community leaders and service providers and fielded a short survey for women of reproductive age in community-based settings such as flea markets, health clinics, and health fairs. Key informant interview participants were asked to refer to the study others they knew who may have self-managed abortion; survey participants who reported attempting self-induction in the past 5 years were also invited to participate.

Research staff contacted eligible women up to three times approximately 2 to 4 weeks after completing their survey or being invited to participate by the ethnographer. Interviews were conducted in English or Spanish by one of three bilingual interviewers trained in qualitative interviewing. Interviews were completed either in-person or by phone and lasted 25–90 min. Participants received a $50 gift card for remuneration. Interviews were digitally recorded and transcribed, and then analyzed in their original language. Representative quotes presented in the results were translated by study author Fuentes.

We developed the interview guide for this study with the objectives of eliciting participants’ narratives of self-managing their abortion and understanding the chronology of their experiences (see Additional file 1). Interview topics included women’s attempts to access clinic-based care, motivations for and experience with self-induction, experience with clinical follow-up, and reflections on the self-induction experience. Data were analyzed using a deductive thematic analysis to describe the trajectory of women’s experiences and their perceptions and assessments of those experiences. Authors Fuentes and Baum coded data using codes developed from interview guide topics and research questions, added new codes to capture themes that emerged from the data (for example, the concept of considering some methods of self-induction “going too far”), and collapsed, expanded, and related the codes by comparing interview narratives within each code to identify and summarize the themes.

We destroyed names and contact information of potential participants when the study was completed. We did not collect signed consent forms to avoid maintaining documentation of participants’ names. To further minimize the risk of a breach of confidentiality, we also obtained a Certificate of Confidentiality from the National Institutes of Health. An information sheet describing the study purpose, procedure, risks, and benefits was offered to each interview participant, the interviewer explained these details, and verbal consent was obtained. The Institutional Review Board of The University of Texas at Austin provided ethical approval for these studies.

## Results

### Sample description

In the 2012 survey, 673 abortion patients were invited to participate, 8 were ineligible, and 318 enrolled, for a 47.8% response rate. In the 2014 survey, we were unable to calculate a response rate for one facility where staff recruited participants (*n* = 57). At the other 9 sites, 624 women were invited; 64 were ineligible and 382 enrolled, for a response rate of 68%. There were a total of 757 survey respondents; 36 did not provide a response to the question about abortion self-induction and were excluded from further analyses. There were no significant differences between study years in the distribution of race/ethnicity, age, language spoken at home, and education level. In the 2014 survey, a smaller proportion of women reported having been born outside the US compared to 2012 (8.9% versus 14.5%).

**Fig. 1:**
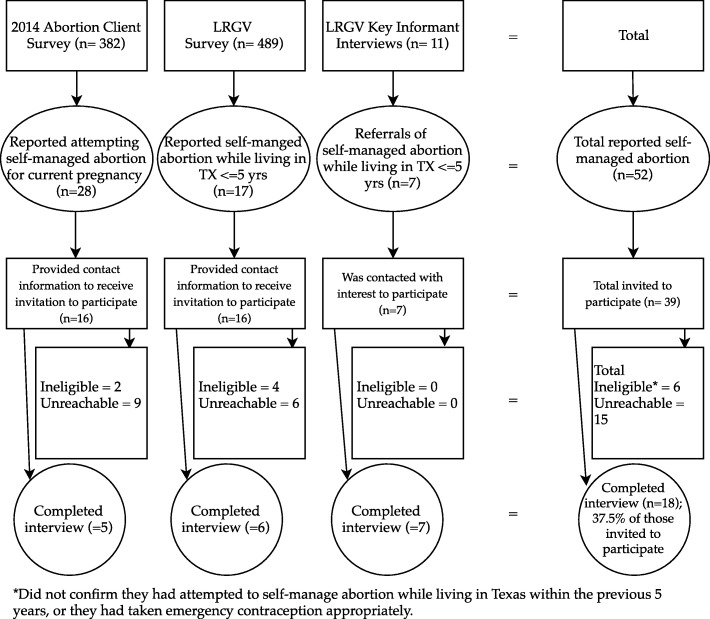
Sources and response rate for qualitative interviews on self-managed abortion among Texas residents

We conducted 18 qualitative interviews; 5 were recruited from respondents to the 2014 abortion patient survey, 6 were recruited from the LRGV survey, and 7 were recruited through the LRGV ethnographic study (See Fig. 1). The ages of respondents ranged from 20 to 42. Seven of the interviews were conducted in Spanish, all of which were with respondents recruited through the LRGV survey or ethnographic study.

Women reported being between 4 and 7 weeks pregnant when they attempted to self-manage their abortion. Nine of the interview participants attempted a self-induction after the implementation of HB 2 in November 2013. Six attempted self-induction before HB 2; five of these took place from 2010 to 2012; one took place in August 2013, after HB 2 had passed but before it was enforced. Two respondents had self-managed abortion in 2013, but it was not clear whether it was before or after HB 2; nevertheless, neither of these respondents attempted to obtain an in-clinic abortion. Finally, one respondent only noted that she attempted self-managed abortion within the time frame for the study criteria, but did not say what year; she reported that she attempted to self-manage her abortion because she did not know where an abortion provider was located.

### Context and reasons for attempting to self-managed abortion

Attempts to self-manage an abortion for their current pregnancy reported by abortion patient survey respondents were, by definition, not successful because they sought clinic-based abortion care subsequently. In the survey, 6.9% (95% CI 5.2–9.0%) of abortion patients reported they had tried to end their current pregnancy on their own before coming to the clinic for an abortion (Table 1). There was no significant difference in the prevalence of attempted abortion self-induction between survey respondents in 2012 (7.3, 95% CI 4.6–10.8%) and 2014 (6.7, 95% CI 4.5–9.5%). Women who reported attempting abortion self-induction were more likely than those who did not report a self-induction attempt to say that it was difficult to get to the clinics (30.0% versus 23.6%) and to live in a Texas county bordering Mexico (15.6% versus 8.1%), although these differences were not statistically significant.

**Table 1:** Characteristics of 2012 and 2014 Texas abortion client survey respondents by attempt to self-manage abortion for this pregnancy

	Total		Attempted to self-manage an abortion for this pregnancy				Fisher’s exact
			No		Yes		
	n	row %	n	row %	n	row %	
Total	721	100	671	93.1	50	6.9	
Year							0.77
2012	303	100	281	92.7	22	7.3	
2014	418	100	390	93.3	28	6.7	
	n	col %	n	col %	n	col %	
Age (*n* = 717)							0.60
18–24 years old	338	47.1	312	46.8	26	52.0	
25+ years old	379	52.9	355	53.2	24	48.0	
Race/Ethnicity (*n* = 712)							0.04
Black	141	19.8	138	20.8	3	6.1	
White	203	28.5	186	28.1	17	34.7	
Latina	298	41.9	276	41.6	22	44.9	
Other	70	9.8	63	9.5	7	14.3	
Language (*n* = 713)							0.20
English Only	568	79.7	532	80.2	36	72.0	
Spanish, Spanish and English, Other	145	20.3	131	19.8	14	28.0	
Residence (*n* = 665)							
Border county	57	8.6	50	8.1	7	15.6	0.08
Non-Border County	593	89.2	557	89.8	36	80.0	
Outside of TX	15	2.3	13	2.1	2	4.4	
Country of birth (*n* = 636)							0.20
US	561	88.2	522	88.3	39	86.7	
Mexico, Latin America or the Caribbean	53	8.3	47	8.0	6	13.3	
Other country	22	3.5	22	3.7	0	0.0	
Gestational age (n = 712)							0.81
< 7 weeks	311	43.7	291	43.9	20	40.8	
7 to 12 weeks	328	46.1	304	45.9	24	49	
13 to 15 weeks	45	6.3	41	6.2	4	8.2	
16 or more weeks	28	3.9	27	4.1	1	2	
Level of ease/difficult traveling to clinic (*n* = 715)							0.31
Easy	543	75.9	508	76.4	35	70.0	
Hard	172	24.1	157	23.6	15	30.0	
Previous abortion (*n* = 693)							0.36
No	411	59.3	379	58.8	32	66.7	
Yes	282	40.7	266	41.2	16	33.3	

There were four primary reasons why qualitative interview respondents tried to self-manage an abortion: 1) they could not afford to get to a clinic or pay for the procedure; 2) their local clinic had closed; 3) a close friend or family member recommended self-induction and 4) to avoid the stigma or shame of having an abortion, especially if they had had prior abortions. No single reason was enough for any participant to consider self-managing their abortion; for all participants, poverty intersected with and layered upon other obstacles to leave them feeling they had no other option.

Almost all of the women had contacted or considered contacting a clinic at some point during their abortion process. They knew about specific abortion clinics from their own prior abortions, information from friends or family, internet searches, or in a couple of cases, just having passed by one in their town. Some women looked into services at a local clinic, but found it had closed or the cost of the procedure was too high. A few women considered clinics farther away, but decided against those options because they were too far or too expensive to travel to. One 20-year-old woman from Houston who described her search for another provider after HB 2 after she discovered that the clinic she had previously been to had closed: “I decided to do a lot of research and I had a lot of options. But the problem was how far the options were and how much they cost.” She attributed her decision to try to self-manage her abortion to the difficulties associated with arranging travel and covering the costs of traveling to a more distant clinic: “… our friend told us about kind of the herbal miscarriage, which she’d done before with someone else. And it was more of a desperate measure thing because of the money and stuff.” Similarly, when another participant was asked if she had tried to use abortion services in Texas, she replied:

I did but was scared -one, because they were asking a lot of money, and at that time I didn’t have a job. So, no I couldn’t – days were going by and then I heard about some pills.” (42-year-old, LRGV)

Other women looked into clinical abortion services after the method they used to attempt to self-manage an abortion did not work; however, their main motivations for attempting to self-manage their abortion were the same as for women who sought clinic-based care before attempting a self-managed abortion: a lack of resources to cover travel and procedure costs. For example, one woman with this experience explained her decision to try to self-manage her abortion after getting pregnant only a few months after her most recent abortion:

I just wanted something to work. I didn’t want to have to spend the money again. I didn’t want to have to do the drive. Not to mention, you know, I don’t have other family. My family lives out of the country so I’m stuck in this town by myself. And my boyfriend I have, but he works, you know, he works and I have to find somebody who’s willing to drive me two and a half hours and back. (26 year-old, Corpus Christi)

Some women perceived stigma or felt shame that also contributed to not wanting to go to a clinic or preferring to keep their abortion decision a secret. For example, the 20-year-old from Houston had borrowed money from a friend for a recent prior abortion, and was embarrassed to ask for financial help again saying, “I didn’t want this to be like a regular thing.”

Of the four women who did not contact a clinic at all, three had previously self-managed abortions successfully with misoprostol. The fourth did not want to go to a clinic where she knew she would face protestors:

I decided on an at-home method for the fact that I didn’t want to be going out to a clinic where I know there’s a lot of protestors or things like that and I didn’t want to be dealing with them telling me that I wasn’t doing the right thing … Even though I did have access … But I guess I didn’t want to tell nobody … I guess I didn’t want to make it more public than what I -- because of all the media and stuff like that about like you shouldn’t abort and things like that. (30 year-old, LRGV)

### Methods of self-managed abortion

Among survey respondents reporting attempted abortion self-induction, the most common method reported was herbs (43.1%) (Table 2). Twelve percent of methods used to attempt to self-manage an abortion was some other drug or medication, 7.8% was misoprostol, and 7.8% was that they hit themselves in the stomach in order to attempt to induce an abortion. Only two women reported using more than one method. Nearly a quarter (21.6%) reported using other methods, including acupressure, heating pad, and papaya preparation.

**Table 2:** Methods of abortion self-management among Texas women

	2012 and 2014 Abortion Patient Survey respondents (*n* = 50)^a^		Qualitative interview respondents (*n* = 18)^a^	
	n	%	n	%
Misoprostol	4	7.8	10	33.3
Other medication	6	11.8	2	6.7
Herbs^b^	22	43.1	11	36.7
Hit abdomen	4	7.8	0	0.0
Vitamin/supplement^c^	2	3.9	5	16.7
Other	11	21.6	2	6.7
No response	2	3.9	0	0.0
Total mentions	51	100	30	100

^a^Survey and interviews respondents could report > = 1 method therefore responses sum to more than number of respondents

^b^Interview respondents reported specifically: Blue cohosh, black cohosh, rue tea, parsley tea, pomegranate rind tea, parsley in the vagina

^c^Vitamin C/caffeine pills

The abortion self-induction methods that qualitative interviews participants used fell into two broad categories: home remedies such as herbs, teas, and vitamins; and medications obtained in Mexico without a prescription (Table 2). All of the women who used medications lived in the LRGV. Women either looked to the internet for information on ways to self-manage an abortion (*n* = 6) or found out about methods from friends or family members. A few reported telling a friend or family member about being unable to afford abortion care who then suggested a self-induction method. Most of those who reported looking online used home remedies to self-manage their abortion, such as herbs, vitamins or food (*n* = 4). Most of those who obtained information from family and friends used medications (*n* = 10).

Women who used home remedies generally searched online for the methods and how to take them, and purchased them at a health food or grocery store, or already had the items in their homes. Two women who used home remedies also tried to obtain misoprostol but were unable to. A 23-year-old from the LRGV who first attempted to self-manage her abortion with herbs then looked online to find the name of the medication used for abortion in clinics; she next asked a friend to buy misoprostol for her in Mexico but her friend was unable to find it at any pharmacy. A 26-year-old who first attempted to self-manage her abortion with home remedies called US pharmacies asking for misoprostol but was told it was by prescription only:

I tried to get a hold of Cytotec, I believe is what it’s called. It’s like the medical abortion pill but they use it for something else. But I called pharmacies and stuff and I tried to ask, you know, “Is this available? Is this available over the counter?” and tried to find out information about it, and that was kind of like a shut down, “Why are you calling us asking about this? … What is this being used for? Why are you asking about it? Do you realize that this is by prescription only?” And eventually I got upset and I was like, “Hey, you know, you are the pharmacist. I’m calling you asking for information … I’m just trying to find out some information and you know, you’re supposed to be able to give me the information I’m asking for.” (26 year old, Corpus Christi)

She then considered trying to buy misoprostol in Mexico but decided not to out of concern that she might obtain counterfeit medications or that it was generally risky.

Women who used medications to self-manage their abortions bought them from pharmacies in Mexico themselves or had a friend or family member go and buy them. Most reported having little or no difficulty finding the medication they were looking for. One 24 year-old woman first attempted unsuccessfully to self-manage her abortion using herbs before she obtained misoprostol. None of the women had a prescription for the medication they were seeking, but most (*n* = 7) knew the medication to be “Cytotec,” and a couple also knew the name “misoprostol.” In some cases, the pharmacy staff person gave instructions about how to take the medication. One woman believed the instructions were incorrect, and she took the medication according to a regimen she learned from searching online and that had worked successfully for her with a previous self-managed abortion.

### Experiences with self-managed abortion and pregnancy outcomes

Of 18 qualitative interview respondents, 10 reported having a complete abortion after taking medications. Eight of these women reported using misoprostol, one woman used hormonal injections, and one took a medication that she said the pharmacists identified as a “steroid”. None of the six women that used home remedies alone reported having a successful abortion.

Women who used misoprostol took various doses and routes of administration. Most described similar abortion experiences, including having intense cramping and then passing large clots. Sometimes women wondered what symptoms were normal. As one woman explained:

It started off slow and … went from zero to sixty real quick and it was just like really painful, intense cramping. It was the worst cramping I’ve ever had and probably one of the worst pains I’ve gone through. And there was also the fact that I’m doing it at home, we’re not – though we have all of the information as to how much bleeding is too much bleeding, you know, or that, there’s always that slight uncertainty of like I don’t really know what I’m doing. (24 year-old, LRGV)

Women confirmed their abortion was complete by seeing a doctor (*n* = 5), or they thought it was complete because they passed large blood clots (*n* = 6). Some women who saw a doctor to confirm completion told their provider that they had self-managed their abortion; two said they told their provider that they had a miscarriage. Some reported that a combination of indicators helped them ensure that the self-managed abortion had worked; for example, a 42-year-old from the LRGV who took misoprostol at home said that she knew the abortion was complete both because she had passed blood clots and because her next period came on time.

Three women did not think they had a complete abortion after taking misoprostol and sought medical care. Of these women, one did not experience any bleeding and subsequently sought abortion care at a Texas clinic. The second experienced cramping and pain, but no bleeding; her husband returned to Mexico for more misoprostol pills and she repeated the process but did not feel it had worked. She decided to continue the pregnancy after a doctor told her she had a healthy pregnancy. The third woman had ongoing bleeding and revealed to her regular gynecologist in Mexico that she had an abortion. She said the provider prescribed birth control pills to complete the process:

She gave me birth control and supposedly by giving me the birth control it was going to make everything come out and that I was going to need a scrape – I believe that’s what it’s called – but at the end, everything came out by itself. It was just the medication, the birth control she gave me …. So after that I didn’t need any procedure. (30 year-old, LRGV)

Six women used herbs, teas, caffeine, seeds, and vitamin C to attempt to self-manage an abortion. They tended to use a combination of methods for 1 to 4 weeks. For example, one woman said she took “basically 3 pills every hour” for more than a week and described her experience as follows:

Yeah, it was just the caffeine that really gave me the symptoms … Oh, I also remember now that I took black cohosh so that’s when I did some research and they said black cohosh with vitamin C would work. And then a special root pill. I can’t remember the name. And after a while taking all the pills was very nauseating and I didn’t want to do it anymore. So, it was just a lot to take in and I wasn’t taking it well, but I kept doing it anyway (20 year-old, Houston)

All six of these women ultimately sought and obtained a surgical procedure when it seemed like their self-induction methods were not working or they worried that the cost of a clinic abortion would increase. For example, one woman who had a previous abortion at her local clinic but found it was closed this time tried to self-manage her abortion unsuccessfully with herbs. When the herbs did not work, she traveled 241 km (150 miles) to an abortion clinic:

I went in it with the best of hope that it [self-induction] would [work], but after a while it was like you know what, this isn’t going to work. It’s going to become …. worried, you know, that it’s too far along, where the price increases, and I was like I’ve just got to get it done now. And I just said well, there’s only like what – I think there’s less than ten clinics in all of Texas now and they’re going to be busy. So when I call to make the appointment, you know, I couldn’t – I think the earliest they saw me was like a month from when I called because they’re so busy, you know. (26 year-old, Corpus Christi)

Some women expressed concern about the safety of the self-induction method they tried; however, no one reported a medical complication as a result of attempting to end their pregnancy on their own, whether they were successful or not. Several women knew of other methods besides the one they used but felt that those methods were “going too far.” In some cases they described a line of risk that they would not consider crossing. For example, one woman who took herbs or vitamins felt that trying to get pills from Mexico was too dangerous. Another woman was willing to get pills from a pharmacy in Mexico, but would not have considered seeking a surgical abortion at a clinic in Mexico. Most participants were unsure of either how safe or effective their method was; they accepted the risks with some fear because they did not see continuing the pregnancy or a clinic abortion as options for them. Exceptions to this were the women who had self-managed an abortion previously. For example, when asked if she ever consulted a doctor regarding her self-managed abortion, a 31-year-old mother of one from the LRGV said “No, because I already knew how to use them so I wasn’t worried about it.”

### Reflections and recommendations after self-managed abortion

Some women worried about the lingering impact of self-inducing abortion on their fertility and well-being. A 30-year-old woman from the LRGV reported that she had not menstruated since her self-induction (using misoprostol) 2 years prior, and she believed that the “miscarriage” caused her period to stop. She was afraid she would not be able to get pregnant again if she wanted to and understood that this was “one of the side effects of having an abortion at home.” The 28-year-old from Corpus Christi also expressed concern that the pills she took (which she described as steroids from Mexico) might have affected her ability to get pregnant:

Actually, I never even investigated on the side effects. I mean, for all I know maybe I can’t get pregnant anymore. I don’t really know what the consequences are. Because now that I’m trying to get pregnant, I don’t know if I’ll be able to.

We asked participants what they would do if they needed an abortion again in the future and what they would say to a friend or family member who needed an abortion. Regardless of the method used or whether it was successful, nearly all participants said that they would go to a clinic and would recommend a friend go to a clinic instead of trying to self-manage their abortion because their abortion self-management experience was difficult, painful, uncomfortable, or frightening (*n* = 5) or because it could be dangerous or may not work (*n* = 4); some did not give a specific reason (n = 4). Even amid the strong expressions that clinic-based abortion services are preferable to trying to do it on one’s own, many acknowledged that in reality women are not weighing a home-based abortion against the comfort, effectiveness, and safety of a clinic-based abortion, but against not having access to care at all because of a lack of money, clinics being too far away, and losing time trying to navigate these challenges. They noted that in the context of women’s real lives, self-induction may be the only or the preferred option. As a 24-year-old from the LRGV said, “I’ve got to say the surgical abortion’s a lot more comfortable so I would rather do it that way if I had to get another one. But money, it is a big deal and 19 dollars is a hell of a lot better than 400.”

Two women who used misoprostol to successfully self-manage their abortions emphasized that they did not envision needing to make a decision about how to end a pregnancy again, but when asked directly what they would do if they did need an abortion again or a friend asked for advice they would recommend “the pills” because they are accessible and they work.

Finally, many women (*n* = 8) also said that they would tell someone seeking an abortion to get information, find out all their options, think about it, and make sure it was their decision, revealing their aversion to making a particular “recommendation” at all. Others noted that they would tell someone to “think twice” and “do their homework” to avoid the risk of regret (in either choosing abortion at all or not choosing it) or that they would support someone in their decision even if it was not what they would do. As one 28-year-old from the LRGV said “I would tell [a friend] to go to a clinic. [She should make sure] that it’s safe, that it’s her decision … But the thing is the lack of money, you know? So then the quickest option are the home remedies or the pills.”

## Discussion

We found that in 2012 and 2014 approximately 7% of Texas abortion patients reported attempting to do something on their own to try to end their current pregnancy before going to an abortion clinic. This is higher than in a 2014 national survey of abortion patients where less than 3% reported ever having tried to end a pregnancy on their own. It is possible that in Texas, where misoprostol can be more easily obtained due to its proximity with Mexico, where there is a large immigrant population from Latin America familiar with self-managed abortion, and where abortion access has been increasingly restricted in recent years, more women know about self-managed abortion and are willing to try to attempt it. For participants in our qualitative interviews, a lack of money, limited transportation, and local clinic closures limited women’s ability to obtain abortion care in a clinic setting and were key factors in deciding to attempt a self-managed abortion. Several women also cited other reasons for attempting to self-manage an abortion, such as feeling shame or stigma about needing a second abortion, or needing an abortion at all; however, this was never the only or primary factor. None of the participants reported attempting self-induction because they preferred it over clinic-based care, with the exception of one woman who acknowledged that although she “had access” to an abortion clinic locally, she self-managed her abortion because she wanted to avoid protestors. This contrasts with a 2008–09 interview study of women in the US who had ever attempted abortion self-induction, in which several participants reported they preferred self-managed abortion because it was more similar to menstrual regulation, more natural, or easier or faster than a clinic-based abortion [3]. Similarly, a qualitative interview study conducted in 2017 of people from 20 states found that in addition to difficulties covering the cost and arranging travel to a clinic, a preference for self-managing abortion over clinic-based services was also represented among the reasons respondents gave for seeking abortion medications online [9].

Texas women who tried to self-manage their abortion used a variety of methods including medications from Mexico and herbs, vitamins, and teas. Most women who used misoprostol used only that method and successfully aborted. Women who used home remedies tended to try various methods in succession over several weeks without success and then sought abortion care at a clinic. These findings are not surprising given the evidence on the high efficacy of using misoprostol to self-manage an abortion, while there is limited evidence supporting the efficacy of other medications and herbs [10]. Prior research has indicated that using ineffective methods for abortion self-induction may contribute to delays accessing clinic-based abortion care, leading women to obtain a procedure later in pregnancy, when it may be riskier and more expensive [3, 11, 12]. Women in our study reported similar experiences; those who used ineffective methods reported doing so for up to several weeks. It is interesting that misoprostol was used by few participants reporting self-managed abortion in the abortion patient survey, possibly because of the high effectiveness of this method. As familiarity with misoprostol increases over time, it is possible that fewer patients will present to clinics after using ineffective methods.

Several women in our study discussed their perception of the dangers of trying to end a pregnancy on their own or their fear of lasting effects on their health or fertility. This added to the stress of the process of their self-managed abortion, a decision that was already made due to a confluence of circumstances that constrained their choices for ending their pregnancy. None of the qualitative interview participants here reported that their self-managed abortion resulted in medical complications; however, we found in the survey of abortion patients that some women did report getting hit in the abdomen to try to end the pregnancy. Little is known about whether women are presenting to emergency departments or other clinical settings with medical complications after abortion self-induction; one study estimated 1.4% of abortion-related emergency department visits between 2009 and 2013 may be due to attempts at self-managed abortion [13].

A limitation of this study is that it is not representative of Texas residents who attempted abortion self-induction in the study period (2009–2014). While we measure the prevalence of attempted self-managed abortion among abortion patients, this clinic-based survey by definition did not measure the prevalence of self-managed abortion among those who did not seek care at a Texas facility. In a previous study we found that 2% of all Texas women reported ever having attempted to self-managed abortion in their life, and 18% of those attempts happened between 2010 and 2015 [2]. Another limitation may be that abortion clients underreported self-managed abortion. Survey underreporting of abortion among non-abortion clients is well-documented [14]; however, we are unable to estimate the possible magnitude of underreporting of self-managed abortion among abortion patients. Nevertheless, our findings most likely represent a conservative estimate of self-managed abortion, indicating that while not common, there is a need to ensure that those who choose to self-manage their abortion have the full resources and information they need to do so. In addition, this analysis excluded 36 survey respondents who did not answer the question about attempted self-managed abortion; therefore, non-response bias may have biased our findings. Finally, our sample size provided limited ability to estimate significant differences in respondent characteristics who attempted to self-manage abortion and not.

Similarly, a limitation of the qualitative interviews is that they represent only some aspects of Texas women’s experiences with attempting self-managed abortion; for example, we found that most women did not attempt to self-manage their abortions because they preferred self-managed care to clinic-based services, but because they could not afford clinic-based care. However, previous research suggests that for some women such preferences could be important factors [3, 9]. Another limitation is that we did not explicitly ask participants if they had fears or concerns about the legal repercussions of attempting self-induction. While this did not emerge as a main theme from women’s narratives, one main concern from clinicians and researchers regarding self-managed abortion is assuring that women are not subject to legal prosecution, particularly as US states continue to pass restrictions on clinic-based abortion services. Indeed, the legal risks of choosing to self-manage abortion may be greater than the medical risk. For example, some women have been prosecuted for allegedly ending their own pregnancies, often under laws that were misapplied because they were intended to protect pregnant women [15]. One key strength of this study is, in addition to describing women’s experiences who eventually sought abortion services at a clinic, we also describe self-managed abortion experiences of women recruited in community settings, several of whom did not access clinic-based care. While these qualitative findings may not capture the full range of experiences and contexts of people in Texas who self-managed abortion during the study period, this study provides novel information about factors that lead to self-managed abortion related to access and information barriers. Future studies on self-managed abortion should assess these experiences in other community-based samples.

In March 2016, the FDA approved an updated mifepristone label to reflect evidence-based practice, making the HB 2 provision restricting medication abortion obsolete [16]. In June 2016, the US Supreme Court decision in *Whole Woman’s Health v. Hellerstedt* found the two provisions of HB 2 responsible for the closing of abortion clinics in Texas to be unconstitutional. However, the great cost and logistical challenges of re-opening clinics that had been shuttered for 3 years meant that, despite these changes, to date only 24 facilities are currently providing abortion services in the state of Texas compared to the 41 clinics that were open before the law was introduced in 2013 [17]. Furthermore, the restriction under HB 15 requiring that women living within 161 km (100 miles) of an abortion facility visit in person at least 24 h before their procedure remains in place. In the 2017 legislative session, Texas passed additional restrictions on abortion care, which are currently enjoined as litigation proceeds [18].

## Conclusion

We suspect that self-managed abortion may become more common if clinic-based abortion care becomes more difficult to access, especially among women in south Texas where misoprostol may be more accessible due to the proximity to Mexico, and among poor women - who make up more than half of all abortion patients [1] and face barriers to accessing reproductive health care. Indeed, a recent study of requests to an online service that provides medication abortion to people living in countries where abortion is legally restricted, and does not provide services to the US, found that three-quarters of the more than 6000 requests from US residents received in a 10-month period in 2017–2018 were from residents of states hostile to abortion [19]. Reproductive health providers can play a key role in supporting the health and well-being of women interested in self-managing abortion for whom clinic-based care is difficult to obtain. Women in our study reported they were concerned during their abortion because they did not have full information or understanding about what to expect, side effects, and long-term health effects. And some women used methods for several days or weeks that ultimately did not work, resulting in delays for some, greater distress, and higher costs. These findings point to a need to ensure that women who may consider abortion self-induction have accurate information about effective methods, what to expect in the process, and where to go for questions and follow-up care. Indeed, there is increasing evidence that given accurate information and access to clinical consultation, self-managed abortion is as safe as clinic-based abortion care [20] and that many women find it acceptable, while others may prefer to use clinic-based abortion care [21].

## Data Availability

The dataset that support the findings of this article belong to the Texas Policy Evaluation Project. At present, the survey data are not publicly available but can be obtained from the authors upon reasonable request and with the permission from the principal investigators KW, KH, DG, and JP. The qualitative data cannot be made available as per the requirement of the Institutional Review Board study approval in order to protect the confidentiality of study participants.

## Abbreviations

ASC: Ambulatory Surgical Center
FDA: Food and Drug Administration
HB: House Bill
LMP: Last Menstrual Period
LRGV: Lower Rio Grande Valley

## Footnotes

1 In 2012 the response category was only “I hit myself in the abdomen”.
